# Financial risk allocation and provider incentives in hospital–insurer contracts in The Netherlands

**DOI:** 10.1007/s10198-022-01459-5

**Published:** 2022-04-12

**Authors:** Chandeni S. Gajadien, Peter J. G. Dohmen, Frank Eijkenaar, Frederik T. Schut, Erik M. van Raaij, Richard Heijink

**Affiliations:** 1 Dutch Healthcare Authority (Nederlandse Zorgautoriteit; NZa), Utrecht, The Netherlands; 2 Rotterdam School of Management, Erasmus University Rotterdam, Rotterdam, The Netherlands; 3 Erasmus School of Health Policy & Management, Erasmus University Rotterdam, Rotterdam, The Netherlands; 4 The Council of Public Health & Society (Raad voor Volksgezondheid & Samenleving; RVS), The Hague, The Netherlands

**Keywords:** Hospital contracts, Financial risk allocation, Purchaser–provider split, Managed competition, Incentives

## Abstract

In healthcare systems with a purchaser–provider split, contracts are an important tool to define the conditions for the provision of healthcare services. Financial risk allocation can be used in contracts as a mechanism to influence provider behavior and stimulate providers to provide efficient and high-quality care. In this paper, we provide new insights into financial risk allocation between insurers and hospitals in a changing contracting environment. We used unique nationwide data from 901 hospital–insurer contracts in The Netherlands over the years 2013, 2016, and 2018. Based on descriptive and regression analyses, we find that hospitals were exposed to more financial risk over time, although this increase was somewhat counteracted by an increasing use of risk-mitigating measures between 2016 and 2018. It is likely that this trend was heavily influenced by national cost control agreements. In addition, alternative payment models to incentivize value-based health care were rarely used and thus seemingly of lower priority, despite national policies being explicitly directed at this goal. Finally, our analysis shows that hospital and insurer market power were both negatively associated with financial risk for hospitals. This effect becomes stronger if both hospital and insurer have strong market power, which in this case may indicate a greater need to reduce (financial) uncertainties and to create more cooperative relationships.

**Supplementary Information:**

The online version contains supplementary material available at 10.1007/s10198-022-01459-5.

## Introduction

Over the past decades, various countries have separated the functions of purchasing and providing care within their healthcare system. The idea behind this purchaser–provider split is that it stimulates competition among providers and creates a means to shift resources to more efficient types of care [46]. In some countries, governments act as purchasers (e.g., regional governments in Spain and municipalities in Finland), while in other countries, health insurers have this role (e.g., in Switzerland, Germany, and The Netherlands). Despite these differences, purchasers and providers across healthcare systems use similar tools to govern their relationship and make (financial) agreements about the provision of care and accompanying conditions. Contracts are one of these tools. The purpose of contracts is to clarify what services are to be provided, at what costs, and under which terms [43]. In addition, they provide a tool to influence provider behavior through the design of financial incentives and allocation of financial risk in provider payment systems [46]. Over the past years, this has become increasingly relevant with the expanding interest in value-based payment reforms to address the issues of suboptimal quality, fragmentation of care, and increasing concerns about financial sustainability [8, 10, 12].

A feature of purchaser–provider contracting that has been particularly challenging is devising, negotiating, and implementing appropriate payment mechanisms with the ‘right’ allocation of financial risk. For example, from theory, it clearly follows that purchasers wishing to maximize incentives for value creation while minimizing socially undesirable behaviors should not transfer all, but only a certain fraction of financial risk to providers [24, 34, 64]. This would imply some form of risk-sharing, in which providers are ideally protected from random and systematic variation in health spending that is beyond their control. However, as the causes of healthcare utilization are often unknown, interdependent, and/or overlapping, this is very difficult to achieve in practice without losing on incentives for value creation. Moreover, financial risk allocation may be affected by for instance the characteristics of providers’ patient populations (a more diverse case-mix is associated with greater financial uncertainty), government policies that create regulatory uncertainty for the provider and/or purchasers, and unbalanced bargaining positions.

There is limited insight into how purchasers and providers in practice allocate financial risk in their contracts. Prior studies on this topic typically do not study the contracts themselves but instead use interviews with those involved in contracting (e.g., [4, 18, 32, 41]). Studies that did look at actual contracts have typically not studied risk allocation explicitly (e.g., [9, 27]. The few studies that did empirically investigate risk allocation in contracts either analyzed the contracts of a single purchaser and/or provider [25, 53], or analyzed all hospital contracts in a country but only for a single year [47]. To our knowledge, no prior studies empirically investigated the determinants of financial risk allocation in hospital–insurer contracts.

Using a unique, nationwide dataset containing almost all contracts concluded between Dutch hospitals and health insurers for the years 2013, 2016, and 2018, this paper aims to identify how insurers and hospitals allocated financial risk in a changing contracting environment (explained in “Changes in Dutch hospital–insurer contracting 2005–2020”). In addition, we analyze possible determinants of financial risk allocation in hospital–insurer contracts. These insights can be used to better understand and design incentives for contracting. The Dutch healthcare system is a particularly interesting contracting environment because of the relatively advanced model of managed competition in which competing insurers negotiate contracts with competing providers [62]. Since the introduction of this model in 2006, the room for contract negotiations has been gradually expanded, particularly since 2012 [19].

This paper is organized as follows. The next section provides an overview of changes in the contracting environment for Dutch hospitals and insurers over the period 2005–2020. The theoretical framework presented in “Theoretical framework” covers the purposes and features of contracts in health care, as well as types of hospital–insurer contracts (characterized by payment method) in relation to financial risk allocation and factors potentially influencing this allocation. The data and methods are described in “Data and methods” and the results are presented in “Results”. Finally, “Discussion” provides a discussion of our main findings as well as some policy implications.

## Changes in Dutch hospital–insurer contracting 2005–2020

The organization and regulation of the Dutch hospital market has changed profoundly during the past decade [19]. Since 2005, hospitals have been paid per Diagnosis Treatment Combination (DTC). DTCs are similar but more comprehensive than diagnosis-related groups (DRGs) that are used in many other countries [40, 66]. Initially, prices were freely negotiable for only a limited number of DTCs. Most prices were derived from budgets, which were negotiated between hospitals and a representative body of all health insurers. To speed up the transition toward individual hospital–insurer contracting, in 2012, several major changes in the payment and contracting system were implemented. First, to reduce the complexity, administrative costs, and incentives for upcoding, the DTC system was simplified by clustering the more than 30,000 DTCs into 4400 new DTC products [30]. Second, the share of the DTC products with freely negotiable prices was doubled from on average 34–70% of hospital revenue. Only for the most complex DTC products, prices are still regulated, though insurers and hospitals are allowed to negotiate lower prices than the maximum prices set by the government (i.e., the Dutch Healthcare Authority). Third, from 2012 to 2014, the prevailing budgeting system was phased out and regulatory budgetary restrictions to hospital production were terminated. This implied that total hospital expenditure could no longer be directly controlled by the government. Therefore, also spurred by the economic recession and the associated necessity to curb public healthcare expenditure, a fourth policy measure was taken. This measure involved the enforcement of so-called “general agreements” between the Minister of Health and the national associations of hospitals, medical specialists, and health insurers to limit the total spending growth of the hospital sector. Initially, for the period 2012–2013, the annual growth limit was set at 2.5% in real terms (i.e., excluding wage and price adjustments), but as a result of growing pressure from the government to contain total hospital expenditure, in subsequent years, the maximum growth rate has been stepwise reduced from 1.5% (2014) to 0% (2022). To be able to enforce these annual growth limits, the government created a “macro control instrument”. This instrument enables the Minister of Health to reclaim any overrun of the agreed-upon maximum total hospital expenditure growth by imposing a levy on each hospital in proportion to its revenue. To date, however, this instrument has not been used in practice, despite total hospital expenditure growth exceeding the agreed-upon limit in 2013, 2016, and 2017 [63]. Finally, in 2015, the remuneration of medical specialists was integrated into the DTC prices. This means that specialists are no longer directly paid by the insurers (a regulated fee per DTC), but by the hospital in which they are active. Since 2015, medical specialists, therefore, have to negotiate their payment share with the hospital board.

## Theoretical framework

### Purposes and incompleteness of contracts in health care

Contracts can serve a variety of purposes [50]. Traditionally, contracts have mainly been viewed as safeguarding mechanisms. As Petsoulas et al. have put it, contracts serve “to minimize uncertainty and to allocate risk between the contracting parties” [41], p. 186). Research has shown, however, that contracts serve purposes beyond safeguarding, including coordination of actions and adaptation to changes that are exogenous or endogenous to the relationship [49, 50]. As a result, contracts typically contain a mix of clauses with provisions for safeguarding, coordination, and adaptation.

Hospital contracting is inherently complex due to the wide array of services provided to many different patient groups. This complexity is difficult to account for in a contract. Furthermore, hospital performance in terms of quality of care and patient outcomes is difficult to measure and, therefore, to capture in contracts. These two features render hospital contracts to be inherently ‘incomplete’ and traditionally rely on fee-for-service and cost-based payment methods, which involve limited financial risk for hospitals. To deal with these contingencies, healthcare purchasers, and hospitals are increasingly investing in and experimenting with alternative provider payment methods [3, 11, 53, 65]. These payment reforms typically expose hospitals to more financial risk associated with medical spending, usually with the aim to enhance incentives for minimizing costs and maximizing quality. Over the past couple of decades, reform efforts with this goal have accelerated with the introduction of bundled and population-based payment contracts, often complemented with risk-sharing and pay-for-performance provisions [8, 10, 58]. A common denominator of these initiatives is that they aim to expose hospitals and other providers to the ‘right’ amount of financial risk to maximize incentives for value creation without unintended consequences.

### Three basic types of hospital–insurer contracts

Three basic contract types can be distinguished, each characterized by different payment methods typically observed in hospital–insurer contracts [20]: (1) open-ended cost-per-case contracts (i.e., without expenditure cap), (2) global budget contracts, and (3) closed-ended cost-per-case contracts (i.e., with expenditure cap). The allocation of financial risk between hospitals and insurers varies substantially between these contract types. More specifically, the contract types differ in terms of allocation of volume risk, i.e., the difference between actual or projected and reimbursed production volume. As price risk (the difference between unit production cost and unit price) is similar across the three contract types, we focus on differences in volume risk. In addition, as explained below, the allocation of financial risk may not only differ between but also within contract types as a result of various ancillary contractual agreements (see Table 1).

**Table 1:** Financial risk allocation for three contract types and associated incentives and ancillary agreements

Contract type by basic payment method	Allocation of financial risk	Positive incentives for hospital	Negative incentives for hospital	Ancillary agreements to reduce negative incentives from basic payment methods
Open-ended cost-per-case contract	Most risk at insurer	Productivity	Overprovision No incentives for quality	Bundled payments Performance-based payments
Global budget	Shared risk between insurer and hospital	Cost containment	Underprovision/quality skimping Risk selection	Performance-based payments Case-mix adjustment Requirement to continue provision of care in case of budget overruns Reimbursement in case of budget overruns Carve-outs Renegotiation in case of budget overruns
Closed-ended cost-per-case contract (with expenditure cap)	Most risk at hospital	Productivity up to certain level Cost containment when cap comes in sight	Overprovision when below cap Underprovision/quality skimping when above cap Risk selection	Performance-based payments Case-mix adjustment Requirement to continue provision of care in case of exceeding cap Reimbursement in case of exceeding cap Carve-outs Renegotiation in case of exceeding cap

Open-ended cost-per-case contracts define the prices per activity, episode or case, and reward providers for volume. As hospital revenues are volume-dependent, this contract type entails a substantial volume risk for the purchasers. Despite DRG-like payment models being introduced to encourage hospitals to increase efficiency, it is known that this contract model can unintendedly encourage hospitals to increase revenues per patient via upcoding and overtreatment, or to increase the number of patients [5, 13, 54]. Hence, open-ended cost-per-case contracts allocate the financial (volume) risk to purchasers, although more recent payment methods, such as bundled payments, can shift some risk to providers (Table 1). Open-ended cost-per-case contracts are especially suitable when volume restrictions are undesirable, like for acute or tertiary care, and can be selectively applied to specific services as part of a comprehensive or master contract [2]. Such ‘carve-out’ agreements are often used as ancillary agreements in global budget or closed-end cost-per-case contracts.

In case of a global budget contract, the hospital receives a guaranteed, prospectively set budget to provide all necessary care to a predetermined population of patients for a fixed period of time [25, 32]. The budget can be based on historical figures, such as healthcare expenditures in the preceding year(s), or on a projection of expected costs based on the expected number of patients, including case-mix adjustments. A global budget implies two-sided risk; the hospital benefits from savings if total claims remain below the budget, but also has to cover the losses if the budget is exceeded. Hospital and insurer thus share the risk, with the exact allocation depending mainly on how ‘tight’ the budget is set and possible ancillary agreements. Global budgets can be preferred if there is a lack of information about or experience with contracting [20], but can also generate waiting lists and incentives to skimp on quality due to the focus on cost control. These drawbacks can be mitigated by ancillary agreements, such as requiring continuation of care provision after the budget is depleted, monitoring performance, and rewarding achievement of desired outcomes [8, 25, 42] (see Table 1). Ancillary agreements can also include renegotiations and/or two-part tariffs with lower payments for care provided after reaching the budget limit.

Closed-ended cost-per-case contracts combine cost-per-case payments with a volume or expenditure cap. This type of contract has also been described as cost-ceiling contract [25]. Purchasers agree to pay per case, activity, episode, or bundle, but only up to a certain amount (the cap). Until that point, payment is volume-dependent. Compared to an open-ended cost-per-case contract, the purchaser’s risk is less as the financial consequences of excess demand or overprovision are borne by the hospital. A closed-ended cost-per-case contract does not oblige the purchaser to pay for services exceeding the cap, but ancillary agreements can be made for such circumstances [20]. As closed-ended contracts allocate most risk to the hospital, they require a high level of maturity in health services planning, control, and cost awareness by the provider. There is a strong incentive to increase production when the volume is below the cap, but also to reduce production as the cap becomes close [7, 35]. As with global budgets, efforts to avoid expenditure overruns can have unintended effects, such as increasing waiting times and quality skimping, which can be prevented or mitigated through ancillary agreements (see Table 1).

Each basic contract model can be combined with elements of performance-based contracting (PBC) with payments being explicitly tied to performance measures on, e.g., accessibility, quality of care, and health outcomes [23, 39, 51]. For example, a bonus could be paid if a hospital meets the criteria of a contractually agreed-upon performance level. Alternatively, the hospital’s share of realized savings (relative to a prospectively agreed-upon spending target or budget) could be made conditional on meeting certain performance targets (e.g., [52]). One of the key challenges in PBC is avoiding the ‘multitasking problem’: as many relevant aspects of performance cannot be measured and thus captured in the contract (rendering contracts incomplete, see “Purposes and incompleteness of contracts in health care”), explicitly paying hospitals for their measured performance may result in a disproportionate focus on the measured aspects of performance [21, 28]. Therefore, the proportion of total hospital payment linked to performance should be limited, underscoring the importance of a careful design of the underlying ‘base’ payment contract and the incentives therein [8, 21, 23].

Finally, the contract period also affects risk allocation and incentives. Long-term contracts reduce uncertainty by providing safeguards for large year-on-year volume and revenue fluctuations. In relationships where one party expects the other to make certain investments (such as implementing value-based health care or setting up network collaborations in a region), long-term contracts will be preferred over short-term contracts [29]. Instead of repeated negotiations and discussions, parties will prefer to agree on ex ante terms and conditions to reduce ex post risks. Long-term contracts encourage more cooperative relationships that might be better suited for achieving long-term strategic objectives of health system improvements [17], such as shifting care from hospitals to ambulatory settings or to home. Moreover, long-term contracts signal mutual trust and may reduce incentives for quality skimping and risk selection, and may also increase incentives for innovation by increasing the time and chances to realize an appropriate return on investment. On the other hand, long-term contracts are relatively inflexible [22, 47] and may create new risks of (financial) agreements that do not fit a dynamic context. Therefore, Crocker and Masten [15] argue that to reduce financial risks, price adjustment processes need to be flexible in long-term contracts in case of uncertain performance over time. Finally, long-term contracts also create a risk of complacency, leading to a lack of incentives for providers to continuously improve performance [55].

### Potential determinants of financial risk allocation

In systems with a purchaser–provider split, purchasers negotiate with providers about the allocation of financial risk in contracts. It is reasonable to assume that both parties (in this paper: insurers and hospitals) will try to negotiate favorable contract terms to serve their own interests, which will include minimizing their own financial risk. Their ability to do so depends on several factors.

A first factor is market power. Health insurers with a strong market power are more likely to negotiate favorable contract terms than insurers with limited market power [45, 60]. Therefore, we expect insurers’ market power to be positively associated with financial risk for hospitals, for example via contracts with expenditure caps instead of global budgets. Similarly, hospitals with high market power are more likely to negotiate favorable contract terms than hospitals with limited market power [16, 31]. Hence, we expect that hospitals with more market power will more often operate under open-ended cost-per-case contracts or contracts with global budgets, than under contracts with expenditure caps.

A second factor is the degree of hospital specialization. For several reasons, more specialized hospitals may be more likely to conclude contracts with less financial risk. First, specialized hospitals tend to treat relatively complex and costly patients. To mitigate the associated risk, both contracting parties may opt for a less risky type of contract. Second, more specialized hospitals are likely to have more market power as fewer hospitals offer the services they provide.

A third potential determinant is the hospital’s financial situation. Particularly, when a hospital experiences financial distress, this may impact financial risk allocation. Hospitals in financial distress will naturally prefer less risky contracts, and insurers may be willing to support this to prevent further deterioration of the hospitals’ financial situation and maintain access to care for their enrollees. Therefore, we expect ‘being in financial distress’ to be negatively associated with the level of financial risk for hospitals in hospital–insurer contracts.

Finally, external factors like national policy changes (e.g., changes in reimbursement systems and budgetary restrictions at the macro-level), are likely to influence financial risk allocation in hospital–insurer contracts.

## Data and methods

Below, we first discuss the data used for our descriptive and regression analyses. Next, we explain the methods for our descriptive contact analysis (“Descriptive analysis”), followed by a description of the regression model used to analyze the determinants of financial risk allocation (“Regression analysis”).

### Data sources

For this study, the actual contracts concluded between Dutch health insurers and hospitals for the years 2013, 2016, and 2018 were made available by the Dutch Healthcare Authority (Nederlandse Zorgautoriteit, NZa). The NZa studies contracts, among other sources, to monitor the Dutch hospital market and to generate evidence for policy recommendations and market regulation. Our data include hospital contracts of the four largest health insurers, representing a combined market share of 88% on average across the 3 years [36, 37]. In the Dutch hospital market, 90, 79, and 75 hospitals were active in 2013, 2016, and 2018, respectively. Table 2 shows that we had access to the contracts of almost all hospitals for each of the four insurers. Overall, 92% of contracts were available over the study period, covering all general and academic/teaching hospitals. The contracts of the remaining hospitals were either not available or deliberately excluded because of the highly distinct case-mix and cost structure of these hospitals. As no more than 2 percent of all contracts in any year were open-ended cost-per-case contracts, we focus our analyses only on contracts with expenditure caps and global budgets. Data for our regression analysis of potential determinants of financial risk allocation were also obtained from the NZa. The NZa maintains a comprehensive and detailed database containing claims data provided by health insurers and data from annual financial reports by hospitals.

**Table 2:** Number and percentage of hospital contracts per health insurer, per year, and total

Health insurer	2013	2016	2018	Total
	*N* (%)	*N* (%)	*N* (%)	*N* (%)
A	83 (92)	71 (90)	75 (100)	229 (94)
B	88 (98)	66 (84)	69 (92)	223 (91)
C	90 (100)	55 (70)	73 (97)	218 (89)
D	88 (98)	74 (94)	69 (92)	231 (95)
Total	349 (97)	266 (84)	286 (95)	901 (92)

### Descriptive analysis

We used a standardized extraction form (see Appendix 1) to extract relevant information on contract type and ancillary agreements as discussed in “Three basic types of hospital-insurer contracts”. A preliminary version of this form was tested on a limited number of randomly selected contracts (*N* = 7). This resulted in minor changes to the form. The final version was used to record the following characteristics: basic payment contract type and the corresponding value in euros, contract duration, and ancillary agreements. Three authors (CSG, PJGD, and RH) extracted data from the contracts using the standardized form. Specifically, one author (CSG) studied all the contracts of 2013 and 2016, and each of the three authors independently studied one-third of the contracts for 2018. The authors regularly worked in the same office space to facilitate discussions and consulted each other in case they had doubts about the interpretation of a specific contract characteristic.

From the extracted data, we calculated descriptive statistics to describe the variation in the use of the basic contract type and ancillary agreements over time and across the four health insurers. Contracts may distinguish between different types of care (e.g., elective versus acute care) and specify different agreements for different types of care. If a contract specified different payment methods for different types of care, e.g., carve-outs, we focused our descriptive analyses on the payment method with the highest contract value in the contract. On average, the dominant payment methods applied to 91% of the contract value.

### Regression analysis

#### Dependent variable

The dependent variable ‘financial risk allocation’ FRA_(*c,h,i,t*)_ reflects the financial risk from the hospital’s perspective of contract *c* between hospital *h* and insurer *i* in year *t*. We categorized contracts into three levels with ascending financial risk for the hospital. The first and lowest level of FRA (1) contains contracts with global budgets, as they are characterized by shared risk between insurer and hospital (“Three basic types of hospital-insurer contracts”). The second, intermediate level of FRA (2) comprises expenditure cap contracts without additional measures or with (mainly) risk-mitigating measures. The third and highest level of FRA (3) involves contracts with an expenditure cap with only risk-enhancing measures.

#### Independent variables

We analyzed the association between financial risk allocation (FRA) and the following explanatory variables: market power insurer, market power hospital, financial distress hospital, degree of hospital specialization, contract year, and insurer. To avoid endogeneity, we used data from the previous year (*t* − 1) to calculate the variables about market power and financial distress.

For each contract, we measured insurer *i*’s market power by *i*’s market share (IMS) in the relevant hospital *h* in the prior year: IMS_(*i*,*h*,*t*−1)_. For each contract, we divided the monetary value of hospital services contracted by insurer *i* from hospital *h* in the prior year by the total value of hospital services of hospital *h* in the prior year. We measured hospitals’ market power by the inverse of the Logit Competition Index (LOCI). LOCI is a competition index that was specifically developed for differentiated product markets such as hospital markets [26]. It depends on hospitals’ market share and observable patient types in each micromarket. Following Berden et al. [6], we used zip-codes to represent the micromarkets. LOCI is 0 under a pure monopoly and 1 in case of perfect competition. By definition, inverse LOCI (invLOCI) is a concentration index. For instance, in case of a duopoly in which the market is equally split between two rivals, LOCI would be 0.5 and invLOCI would be 2. The higher invLOCI, the higher the market power of hospitals. We used data from hospital micromarkets in the prior year to calculate the concentration index for each hospital *h*: invLOCI_(*h*,*t*−1)_. Hospital financial distress (HFD) was measured using the solvency rate of each hospital, defined by net assets divided by total debt in the prior year. In the contracts, we observed that a solvency rate below 8% is typically seen as a risk in hospital–insurer contracts. Therefore, we defined HFD as a solvency rate below 8%. We distinguished two categories of hospitals to indicate the degree of hospital specialization (DHS): general hospitals and academic/teaching hospitals. For each of the four insurers, a dummy variable is included (IB, IC, ID with IA as reference). Finally, to control for external policy and regulatory changes as described in “Changes in Dutch hospital-insurer contracting 2005–2020” and “Potential determinants of financial risk allocation”, we included dummy variables for contract years 2016 and 2018 (with year 2013 as reference).

#### Regression model

We used ordinal mixed logistic regression modeling to analyze the association between FRA and the independent variables described above. The model contained a random hospital intercept (*μ*) to account for the clustering of contracts in each hospital. All other variables were modeled as fixed effects. We added the interaction variable IMS*invLOCI to test whether there is a combined effect of these variables on FRA. The regression equation of the model is as follows, with *j* representing the ordinal level of FRA:﻿

$$\begin{aligned} & {\text{Log}}\left( {\frac{{P({\text{FRA}}_{(c,h,i,t)} \le j}}{{P({\text{FRA}}_{(c,h,i,t)} > j}}} \right) = \beta_{0} + \beta_{1} {\text{IMS}}_{(i,h,t - 1)} + \beta_{2} {\text{invLOCI}}_{(h,t - 1)} + \beta_{3} {\text{IMS}}_{(i,h,t - 1)} *{\text{invLOCI}}_{(h,t - 1)} + \beta_{4} {\text{HFD}}_{(h,t - 1)} + \beta_{5} {\text{DHS}}_{(h,t - 1)} + \beta_{6} {\text{Year}}2016 + \beta_{7} {\text{Year}}2018 + \beta_{8} {\text{IB}}_{(c,h,i,t)} + \beta_{9} {\text{IC}}_{(c,h,i,t)} + \beta_{10} {\text{ID}}_{(c,h,i,t)} + \mu_{(h)} + \varepsilon_{(c,h,i,t)} \\ \end{aligned}$$

## Results

### Descriptive analysis

#### Basic contract type

**Fig. 1:**
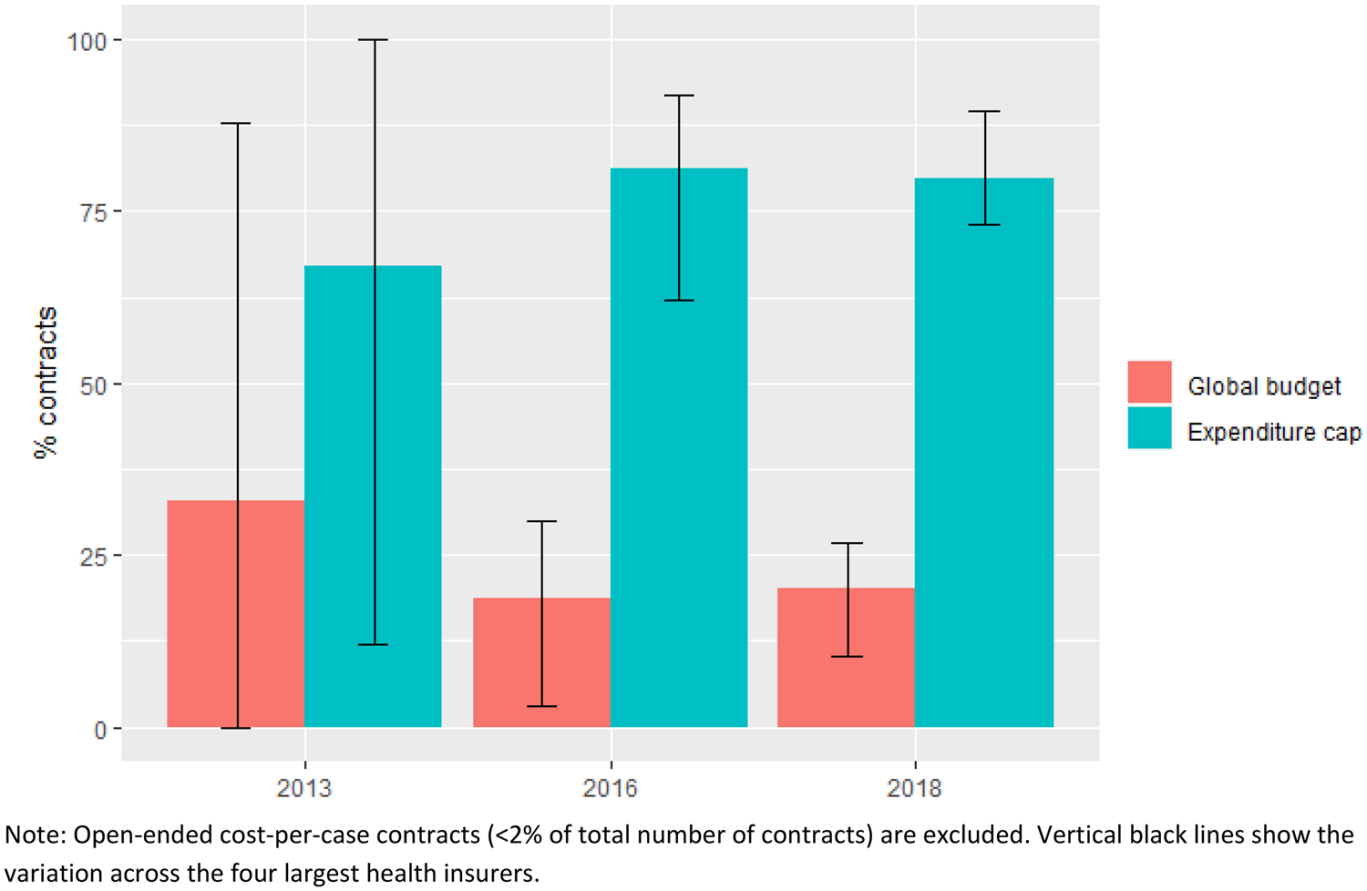
Contracts with expenditure cap and global budget as a percentage of all contracts, 2013–2018

As mentioned, the vast majority of contracts were either global budget or closed-ended cost-per-case contracts (i.e., with expenditure caps). Both contract types were typically complemented with ancillary agreements to mitigate possible negative incentives and adjust risk allocation. Figure 1 shows that the share of contracts with expenditure caps increased over time, from 67% of the total number of contracts in 2013 to 81 and 80% in 2016 and 2018, respectively. The share of contracts with a global budget decreased from 33% in 2013 to 20% in 2018. Figure 1 also shows that the variation across insurers in the use of global budgets and expenditure caps (as indicated by the vertical black lines) decreased over time; while in 2013, there was at least one insurer with only expenditure caps and hence no global budgets at all, in 2018, all of the four largest insurers used a mix of these two contract types.

#### Carve-outs

Carve-outs were used in contracts with expenditure caps and global budgets. These carve-outs included specific types of care, such as expensive drugs and highly complex care. Over the years, expensive drugs were increasingly carved-out from the basic contract type and paid on a separate open-ended cost-per-case basis. The size (in euros) of these carve-outs was not specified in most contracts. In case the size of carve-outs was specified, it entailed a small part of the total contract value.

#### Ancillary agreements

**Fig. 2:**
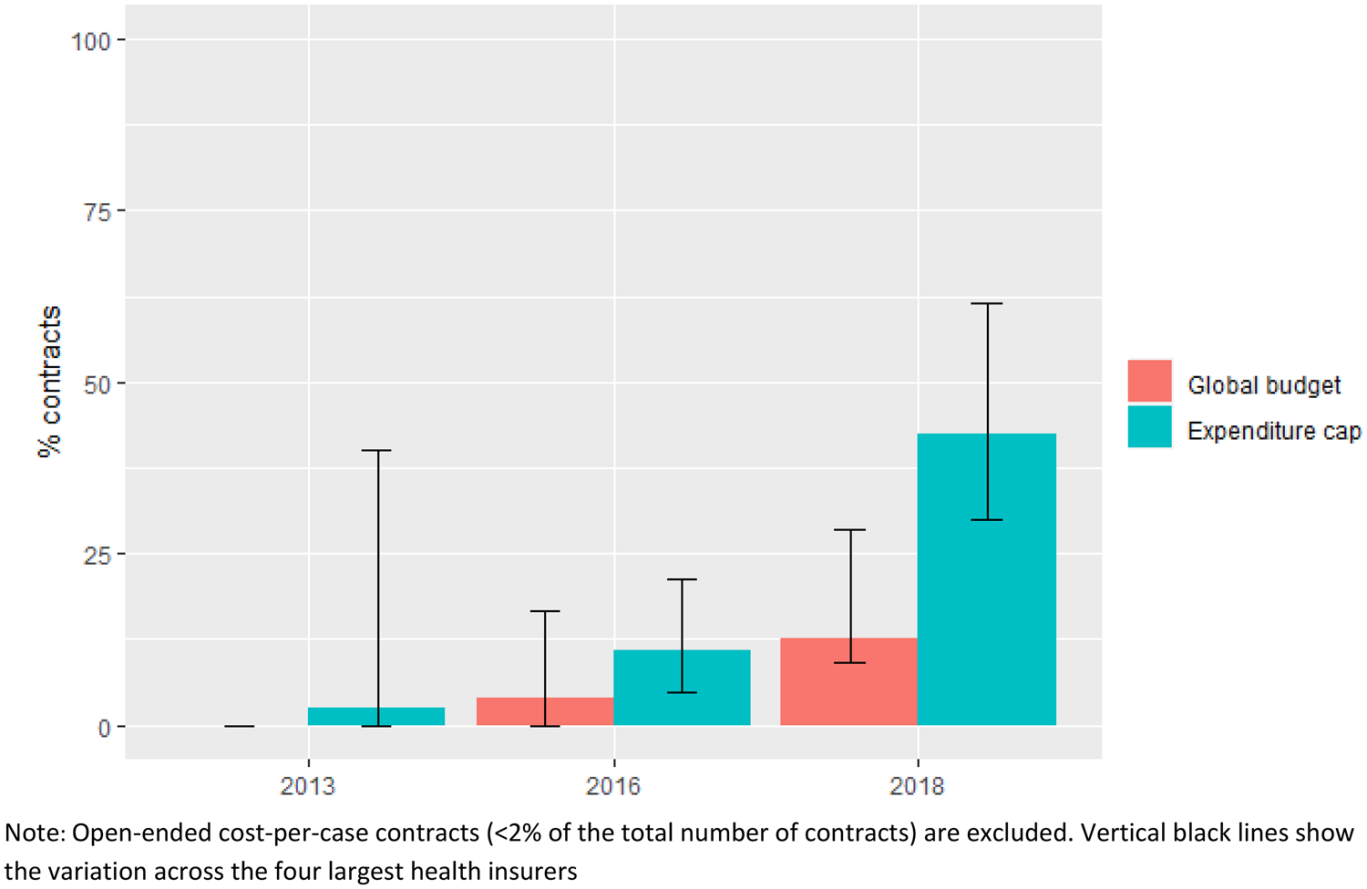
Contracts with an agreement to (partly) reimburse care in case of cap or budget overruns as a percentage of all contracts of the relevant type, 2013–2018

As shown in Fig. 2, the share of expenditure cap contracts with an agreement to (partly) reimburse care after reaching the cap increased from 11% in 2016 to 42% in 2018. This risk-mitigating measure was much less common in global budget contracts. Two main applications of this measure were used: two-part tariffs and “doughnut holes”. Two-part tariffs reimburse additional care at a lower or diminishing rate compared to the standard price. Doughnut holes specify a gap in reimbursement that starts and ends at a certain level of hospital expenditures. In 2018, 60% of all partial reimbursement agreements involved two-part tariffs only. The remaining 40% consisted of a combination of two-part tariffs and doughnut holes. The exact operationalization of two-part tariffs and doughnut holes, in terms of percentages and levels of expenditure, varied across contracts.

Another way to reduce financial risk for the hospital is to allow for renegotiating the originally agreed-upon cap or budget level. Most of the contracts of three insurers in 2013 and two insurers in 2016 and 2018 included such an agreement in general terms. The contracts specified under which conditions renegotiation could take place, generally described as extraordinary circumstances, a clear risk of exceeding the cap or budget, or external causes of a cap or budget overrun. To prevent hospitals from refusing to admit and treat patients once the agreed-upon cap or budget has been reached, three out of four health insurers in most contracts included a requirement to continue the provision of care in case of exceeding the cap or budget (on average 89% of the contracts, ranging from 76 to 97% across the three insurers in 2018). The share of contracts with such a clause was quite stable over the years.

#### Performance-based rewards and bundled payments

Hardly any contract included agreements on explicit financial rewards for quality of care. Of all 2018 contracts, only 5% included shared savings agreements aimed at rewarding cost control. These agreements either pertained to specific projects or types of care, such as expensive drugs or cardiac care, or to savings resulting from staying below the cap or budget. One insurer used bundled payments for specific medical conditions (e.g., cataract and breast cancer) in its 2018 contracts with 22% of the hospitals. These bundled payments typically cover all hospital care within 2 years after surgery for these specific medical conditions and in most cases, there was no maximum number of bundles that could be claimed (i.e., no cap). In case the hospital maintained or improved quality, the agreement would usually be extended. In addition, all insurers participated in a national bundled payment experiment for maternity care. This is an ongoing, voluntary experiment in which several hospitals participate and which pertains to both primary and hospital care [44].

#### Long-term contracts

**Fig. 3:**
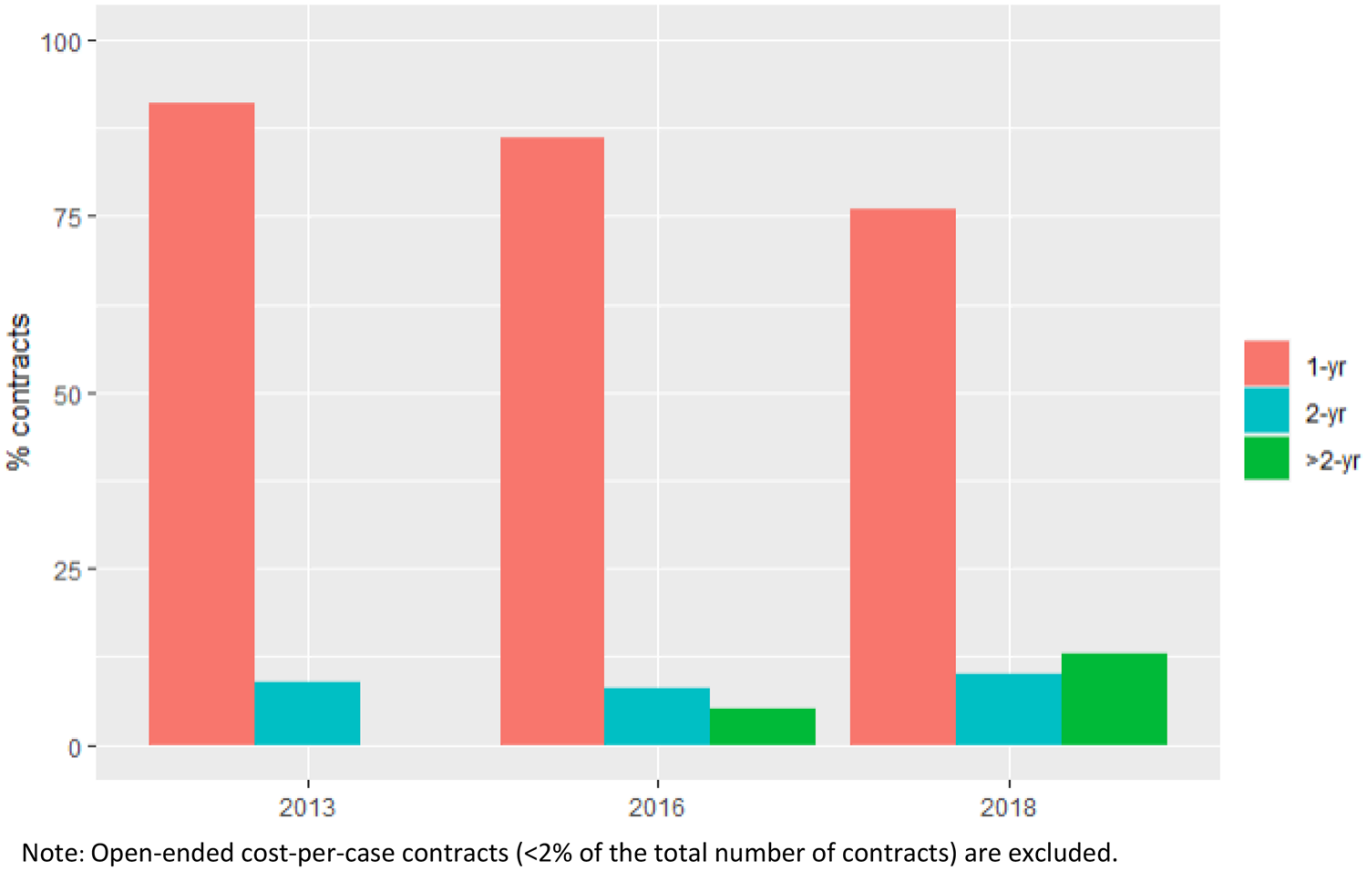
1-year, 2-year, and > 2-year contracts as percentage of all contracts, 2013–2018

As shown in Fig. 3, the share of 1-year contracts reduced from 91% in 2013 to about 76% in 2018, while the share of contracts with a duration of 3 or more years increased from 0% in 2013 to 13% in 2018.

Focusing on 2018, Fig. 4 shows that in contrast to 1-year contracts, the majority of long-term contracts included a global budget. Also in 2016, the share of global budgets was greater in multiyear contracts (36%) compared to 1-year contracts (16%).

**Fig. 4:**
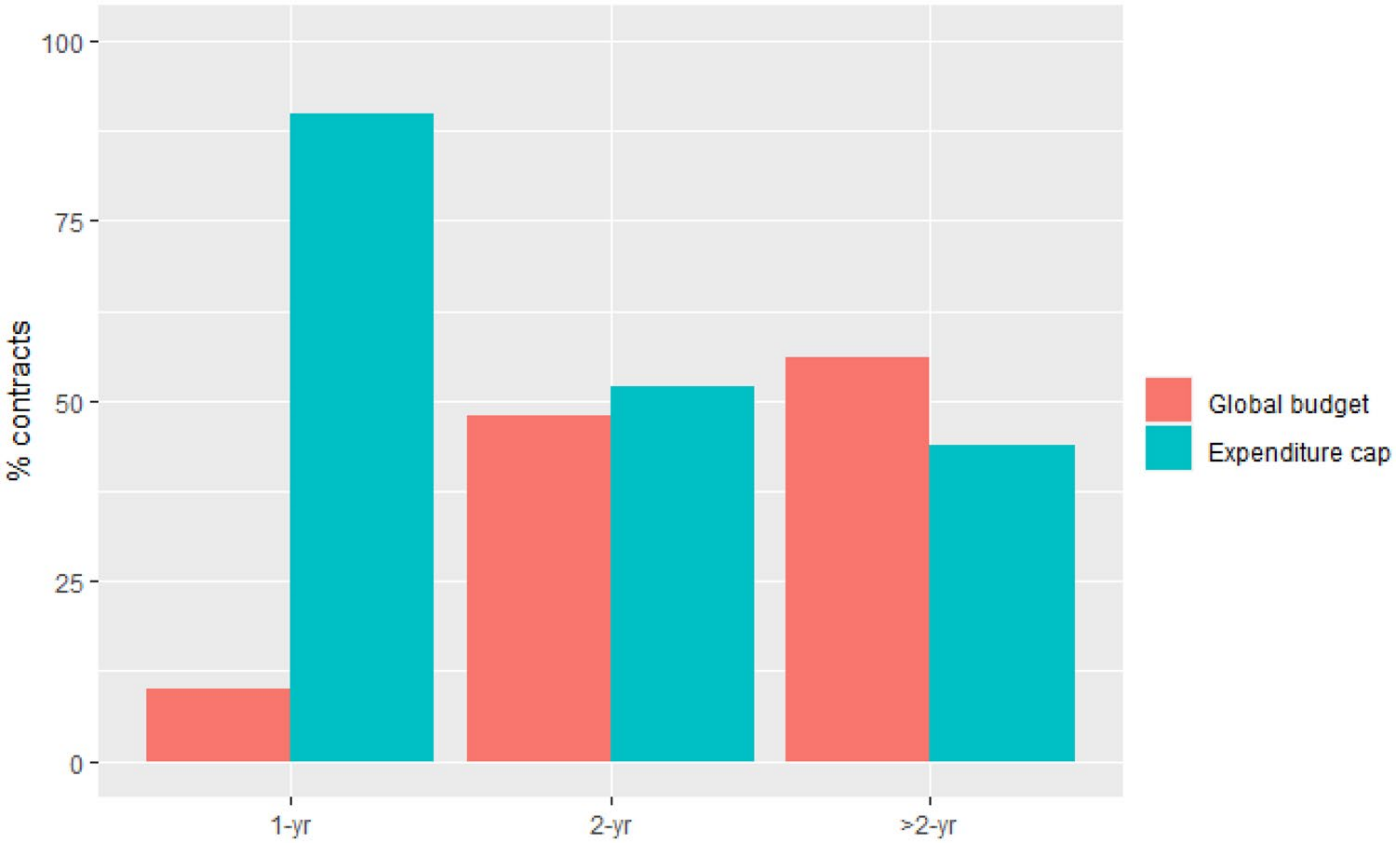
Expenditure cap and global budget contracts as percentage of 1-year, 2-year, and > 2-year contracts, 2018

### Regression analysis

#### Descriptive statistics

Table 3 shows descriptive statistics for key variables included in the regression model. Overall, the level of financial risk for hospitals (FRA) increased from 2013 to 2016. Between 2016 and 2018, the number of high-risk contracts was reduced in favor of the number of contacts with intermediate risk. The average market share of health insurers (IMS) remained stable over time, while the average market power of hospitals (invLOCI) decreased slightly. Finally, the percentage of hospitals under financial distress (HFD) decreased on average from 5% in 2013 to 2% in 2018.

**Table 3:** Descriptive statistics of key variables in the regression model

Year	2013	2016	2018
Contracts (*N*)	339	262	276
FRA (*N* (%))			
Low risk	112 (33%)	49 (19%)	55 (20%)
Intermediate risk	86 (25%)	119 (45%)	147 (53%)
High risk	141 (42%)	94 (36%)	74 (27%)
IMS (mean, SD)	0.22 (0.19)	0.22 (0.17)	0.22 (0.18)
invLOCI (mean, SD)	2.18 (0.80)	2.12 (0.69)	2.04 (0.58)
HFD (*N* (%))			
SR lower than 8%	12 (5%)	12 (5%)	6 (2%)
SR 8% or higher	254 (95%)	242 (95%)	263 (98%)
DHS (*N* (%))			
General	213 (63%)	148 (56%)	151 (55%)
Top clinical/academic	126 (37%)	114 (44%)	125 (45%)

*FRA* financial risk allocation, *IMS* insurer market share, *invLOCI* inverse logit competition index, *HFD* hospital financial distress, *SR* solvency rate, *DHS* degree of hospital specialization

#### Regression results

Table 4 shows the results of the ordinal logistic regression analysis. The model shows a statistically significant association between the dependent variable financial risk allocation (FRA) and insurer market power (IMS), hospital market power (invLOCI), the interaction between insurer and hospital market power (IMS*invLOCI), year (2016), and insurer B, C, and D. The results show that both a higher market share of an insurer in a hospital (IMS) and the indicator of hospital market power (invLOCI) are significantly associated with a lower level of financial risk for the hospital. As indicated by the OR, a 1 unit increase of the invLOCI results in a 0.45 times higher odds of an intermediate and high level of FRA (i.e., contracts with expenditure caps) compared to the odds of the lowest level of FRA (i.e., contracts with global budgets). The effect of the interaction variable IMS*invLOCI is significant and positive, which indicates that both variables strengthen each other’s (negative) effects on FRA. The results for the year dummies show that only 2016 is significantly related to FRA: the odds of an intermediate and high level of FRA is 1.72 times higher than the odds of the lowest level of FRA in 2016 compared to 2013. The effect of the dummy for 2018 is not statistically significant (relative to reference year 2013), which can be explained by the increasing use of risk-mitigating measures between 2016 and 2018 (see e.g., Fig. 2). Compared to the reference insurer A, the other three insurers (B, C, and D) are more likely to sign contracts with more financial risk for hospitals. This is especially the case for insurer B (OR = 35.50).

**Table 4:** Ordinal logistic regression results of the determinants of financial risk allocation

	Coefficient	Standard error	Odds ratio	*p* value
IMS	− 6.573	1.557	0.00	0.000
invLOCI	− 0.797	0.208	0.45	0.000
IMS*invLOCI	1.948	0.640	7.01	0.002
2016	0.540	0.190	1.72	0.004
2018	0.005	0.184	1.00	0.980
Insurer B	3.569	0.261	35.50	0.000
Insurer C	1.039	0.233	2.83	0.000
Insurer D	0.650	0.198	1.92	0.001
HFD	0.624	0.465	1.87	0.180
DHS	− 0.164	0.185	0.85	0.377

*FRA* financial risk allocation, *IMS* insurer market share, *invLOCI* inverse logit competition index, *HFD* hospital financial distress, *DHS* degree of hospital specialization

## Discussion

In this paper, we have analyzed how Dutch health insurers and hospitals allocated financial risk in their contracts over the period 2013–2018. In addition, we explored the association between financial risk allocation and market power (of hospitals and insurers) and other potential determinants.

### Summary of results

The following results stand out from our analysis. First, the share of contracts with an expenditure cap increased from 67% in 2013 to 80% in 2018, with a concomitant reduction of the share of contracts with a global budget. This suggests that hospitals were exposed to more financial risk over time, although this increase was counteracted by an increasing use of risk-mitigating measures between 2016 and 2018. During the study period, open-ended cost-per-case contracts were virtually absent in the Dutch hospital market. Second, performance-based agreements were hardly used throughout the study period. Third, multiyear contracts were increasingly used over time, and were especially used in combination with global budgets. Fourth, financial risk allocation was associated with (the interaction between) insurer market power and hospital market power, and health insurer.

### The role of market power

In line with our expectations, a stronger market position of hospitals—relative to other hospitals—is associated with less financial risk for the hospital. Contrary to our expectations, however, health insurers seemed to prefer contracts with lower financial risk for a hospital if they had a higher market share in that hospital. Hence, insurers do not appear to use their market power to shift more financial risk to the hospitals. Rather, they seem to prefer more financial certainty for these hospitals. This effect is larger when hospitals have more market power, which may reflect the strong mutual dependency and a related desire for more cooperative relationships, and a shared long-term ambition. This explanation is supported by previous work showing that insurers conclude multiyear contracts with global budgets, particularly in regions where they have a high market share [38]. Such contracts signal trust and could provide better conditions for long-term investments in innovation and efficiency (e.g., substitution of hospital care to cheaper but equally effective alternatives) as financial uncertainties are reduced [14, 38]. Finally, our results show that, independent of market power, health insurers had quite different contracting strategies. This suggests that variation in purchasing policies and negotiation skills across insurers results in a different allocation of financial risk toward hospitals.

### The impact of national policies following the economic recession

The worldwide economic recession from 2008 to 2012 created an urgency to enforce cost control policies in The Netherlands, like in most other European countries [59]. From 2012 onwards, the Dutch Minister of Health, therefore, concluded “general agreements” with national associations of hospitals, medical specialists, and health insurers in which national maximum annual growth rates for total hospital expenditures were defined. Even though these agreements were not strictly binding for local negotiations, our results indicate that they did translate into the use of cost control measures, specifically global budgets and expenditure caps, in hospital–insurer contracts. Over time, these national maximum growth rates were lowered (from 2.5% in 2012 to 1.5% in 2014, to 0.8–0.0% in 2019–2022), which seems to have been translated into a shift of financial risk toward hospitals, mainly between 2013 and 2016. This shift may also be related to the profound changes in the hospital payment system since 2012, which created uncertainty about the appropriate prices for (new and non-regulated) DTC products and their impact on hospital spending [19].

### Performance-based agreements

Our results indicate that health insurers have predominantly focused on cost control. This may not only be due to the pressure by the government to limit hospital expenditure growth at the national level, but also by a strong focus on price competition between health insurers. This focus on price competition is at least partly due to the fact that competition on quality is risky and difficult given the lack of transparency of hospital quality [56]. Even though many quality registries exist (mostly for specific diseases) and a wide variety of indicators have been developed, it still appears very difficult to mutually agree on a set of quality measures that can be used in hospital-level contracts. Insurers are experiencing disincentives to use quality measures in their purchasing policies since they fear this might provoke strong provider opposition, which may damage their reputation [57].

These findings also show that attempts of the government to stimulate performance-based payment and contracting did not have a substantial impact. In 2011, members of the Dutch parliament requested the Minister of Health to develop a vision and plan for the introduction of “outcome-based payments” in the Dutch healthcare system by 2020 [61]. The Minister of Health embraced this ambition, and in the following years, the government has attempted to facilitate the transition to outcome-based payment through the establishment of an independent Healthcare Quality Institute (2014) to develop quality standards, and by encouraging local payment reform experiments and development of outcome indicators. Nevertheless, our results indicate that outcome-based payment models are still hardly being used in Dutch hospital care.

### Impact of COVID-19

Recently, the COVID-19 crisis has created concerns about increased financial risks for hospitals due to uncertainty about current and future costs of hospital care. For the years 2020 and 2021, insurers decided to transform all contracts with hospitals into a global budget covering the additional investments that hospitals needed to make to cope with the pandemic as well as foregone revenues related to the postponement of regular care [67]. Because insurers and hospitals agreed that no hospital would have to make a loss because of the pandemic, the resulting financial risk was largely borne by the insurers. At the same time, insurers have agreed to share these losses and decided that no single health insurer should experience a disproportionate loss as a result of these compensations [1]. Moreover, insurers are largely compensated by the government on the basis of a “catastrophe clause” in the Health Insurance Act. At this moment, it is unclear what the impact of the COVID-19 crisis will be on financial risk allocation in future years.

### Strengths and limitations

A main strength of this study is that it used unique data covering almost all contracts between Dutch hospitals and health insurers over multiple years, as well as information on potential determinants of financial risk allocation. In addition, the Dutch hospital sector presented an interesting case for analyzing financial risk allocation in these contracts because of the role of health insurers as prudent buyers of care and new government policies changing the contracting environment.

Nevertheless, our results should be interpreted with the following limitations in mind. First, we defined financial risk on the basis of the main type of payment contract. We did not have information on hospital costs (in terms of labor and capital) and on how global budgets or expenditure caps were actually determined. Therefore, we were unable to assess how restrictive the expenditure caps or global budgets were in practice. In other words, we were not able to determine the *exact size* of the financial risk hospitals were actually exposed to. Nonetheless, we believe that the results do show the *direction* of changes in financial risk over time. Second, we did not study how hospitals and health insurers actually dealt with potential budget overruns during the contract year and to what extent ancillary agreements about the continuation of care, partial reimbursement of additional care, and renegotiation of budget ceilings or expenditure caps were used in practice. Finally, our statistical model likely suffers from omitted variable bias; it is likely that there are other factors that influenced financial risk allocation, which we could not observe.

### Implications

These limitations notwithstanding, our findings suggest that without additional measures, national policy goals are not automatically translated into local contract negotiations. Although the general agreements between the government, hospitals, and health insurers appear to have contributed to the goal of cost control at macro-level through contracts, at the same time other elements of these agreements (e.g., on substitution of care) have had a much smaller impact on contracting [48]. Furthermore, insurers currently lack incentives to work toward the national goal of performance-based contracting, and hospitals lack incentives to invest in transforming the organization of care. Our findings imply that enhancing balanced market positions seems to stimulate cooperative relationships needed for performance-based contracting and long-term investments. This should be accompanied by better and more transparent information about the quality of care.

In addition, policymakers should consider providing more (local) guidance and/or regulations to reach performance-based contracting, e.g., through sharing best practices. This should facilitate and encourage healthcare purchasers and providers to realize more contracting maturity and invest in mutual trust and long-term relationships with a shared vision and ambition. As mentioned, we have already seen several examples of successful long-term contracting with concomitant improvements in care.

Future research could provide better insights into how to achieve higher levels of contracting maturity, including other ways to allocate financial risk. One approach would be to study the contracts of one hospital–insurer combination over multiple years (e.g., [33]). In addition, further research into the process of designing, negotiating, and implementing a contract could enrich our understanding and interpretation of the content of the types of contracts which we have analyzed. Finally, we suggest monitoring how contracting develops in the aftermath of the COVID-19 crisis, as it will provide new insights on the influence of exogenous shocks and uncertainties on risk allocation between purchasers and providers of health care.

## Supplementary Information

Below is the link to the electronic supplementary material.

Supplementary file1 (XLSX 17 KB)

## Data Availability

The Dutch Healthcare Authority has access to the research data. Due to the confidentiality of data, the data are not available to third parties.
